# Molecular Characterization, Expression Analysis, and CRISPR/Cas9 Mediated Gene Disruption of Myogenic Regulatory Factor 4 (MRF4) in Nile Tilapia

**DOI:** 10.3390/cimb46120820

**Published:** 2024-12-04

**Authors:** Zahid Parvez Sukhan, Yusin Cho, Shaharior Hossen, Doo Hyun Cho, Kang Hee Kho

**Affiliations:** Department of Fisheries Science, Chonnam National University, Yeosu 59626, Republic of Korea; zpsukhan@jnu.ac.kr (Z.P.S.); choys@jnu.ac.kr (Y.C.); shaharior.pstu@gmail.com (S.H.); chodoo@jnu.ac.kr (D.H.C.)

**Keywords:** growth, myogenesis, MRF4, myf6, CRISPR/Cas9, Nile tilapia

## Abstract

Myogenic regulator factors (MRFs) are essential for skeletal muscle development in vertebrates, including fish. This study aimed to characterize the role of *myogenic regulatory factor 4* (*MRF4*) in muscle development in Nile tilapia by cloning *NT-MRF4* from muscle tissues. To explore the function of *NT-MRF4*, CRISPR/Cas9 gene editing was employed. The *NT-MRF4* cDNA was 1146 bp long and had encoded 225 amino acids, featuring a myogenic basic domain, a helix-loop-helix domain, and a nuclear localization signal. *NT*-MRF4 mRNA was exclusively expressed in adult muscle tissues, with expression also observed during embryonic and larval stages. Food-deprived Nile tilapia exhibited significantly lower *NT-MRF4* mRNA levels than the controls while re-feeding markedly increased expression. The CRISPR/Cas9 gene editing of *NT-MRF4* successfully generated two types of gene disruption, leading to a frame-shift mutation in the NT-MRF4 protein. Expression analysis of *MRF* and *MEF2* genes in gene-edited (GE) Nile tilapia revealed that *MyoG* expressions nearly doubled compared to wild-type (WT) fish, suggesting that *MyoG* compensates for the loss of MRF4 function. Additionally, *MEF2b*, *MEF2d*, and *MEF2a* expressions significantly increased in GE Nile tilapia, supporting continued muscle development. Overall, these findings suggest that *NT-MRF4* regulates muscle development, while *MyoG* may compensate for its inactivation to sustain normal muscle growth.

## 1. Introduction

Muscle myogenesis is a series of events involved in muscle development. The initiation and regulation of these processes are governed by myogenic regulatory factors (MRFs). The MRF gene family comprises four members of muscle regulatory proteins including myoblast determination protein (MyoD), myogenic regulatory factor 5 (MRF5), also referred to as myogenic factor 5 (myf5) (hereafter referred to as MRF5), myogenic regulatory factor 4 (MRF4), also known as myogenic factor 6 (myf6) (hereafter referred to as MRF4), and myogenin (MyoG) [1,2,3,4,5]. The first protein identified in the MRF family was MyoD [2]. The other three MRF proteins were discovered shortly after and were found to share homology with MyoD [3,4,5]. MRF4 was the last of these to be identified [5]. MRF family proteins possess a basic helix-loop-helix (bHLH) domain, enabling them to recognize the E-box motif (CANNTG) in the regulatory regions of target genes. These E-box motifs are commonly located in the regulatory regions of genes expressed by the E-protein family of bHLH proteins [6]. The bHLH domain exhibits high homology among MRF family proteins, enabling similar roles in muscle development. MyoD and MRF5 play key roles in differentiation and proliferation during embryogenesis, while MyoG and MRF4 primarily drive myoblast differentiation [1].

MRFs demonstrate almost similar functions across higher vertebrates [1] and fish [7]. However, both extrinsic and intrinsic factors must be considered when studying growth-related genes in fish [8]. Extrinsic factors, such as temperature and food supply, and intrinsic factors, including age, sex, genetics, and gene interactions, influence muscle growth in fish [9]. The muscle regulatory gene *MRF4* is expressed in various adult muscle fibers [10] and its expression is reported to be the highest among the MRFs in adult rodent muscle [11]. However, the specific role of MRFs remains to be determined due to gene interactions at the complex MRF4/MRF5 locus, where the effects of one gene are influenced by other genes [12]. Although the regulation of MRF4 has been extensively studied in higher vertebrates, particularly in rodents [5,13], research on MRF4 in fish remains limited. Notable exceptions include studies on zebrafish (*Danio rerio*) [14], yellowtail amberjack (*Seriola lalandi*) [15], common snowtrout (*Schizothorax richardsonii*) [16], and ya-fish (*Schizothorax prenanti*) [17]. *MRF4* expressions have previously been reported to vary across different developmental stages [15,17] and food-deprived fish [16]. Although some studies have functionally characterized MRFs by producing MRF mutant offsprings [13,18,19]; however, no published reports have used CRISPR/Cas9 techniques to investigate *MRF4* in fish.

CRISPR/Cas9 is an effective and straightforward genome editing technology that can efficiently generate specific gene disruptions. The CRISPR/Cas9 system comprises the Cas9 endonuclease and a single guide RNA (sgRNA), which directs the Cas9 enzyme to a particular location within the genome. Once at the target sequence, Cas9 cleaves the DNA, resulting in double-strand breaks that enable precise modifications to the genome. This technique has been extensively applied to generate frameshift mutations in coding protein sequences, which resulted in the loss of functional alleles in many fish species including Nile tilapia (*Oreochromis niloticus*) [20]. Although CRISPR/Cas9 is a popular technology for producing progeny with enhanced growth and immunity, it is now extensively used for the functional characterization of genes [21,22,23,24]. In the present study, gene editing was performed on the *MRF4* gene in Nile tilapia to facilitate its functional characterization.

Nile tilapia is a highly versatile fish and is often called the “aquatic chicken” due to its adaptability and widespread consumption. It originates from tropical and subtropical Africa, and is now farmed worldwide, including in South Korea [25]. It is not only useful for aquaculture, but also a key non-model species for research in various scientific fields: growth and physiology [26], endocrinology [27], genomic biology, and molecular genetics [28]. Despite genetic improvement programs aimed at enhancing tilapia growth in Asian countries, limited genetic diversity, inbreeding, and hybridization have resulted in decreased growth rates and earlier sexual maturation in farmed populations. Therefore, the potential role of environmental factors, age, sex, and genetics on the growth and development of tilapia have been extensively studied [29,30,31,32]. However, the molecular mechanism through which MRFs function in development and muscle regulation has yet to be explored. Consequently, this study characterized a muscle regulatory gene, *MRF4*, in Nile tilapia. An extensive in silico analysis of Nile tilapia *MRF4* was conducted and analyzed mRNA expressions in different experimental tissues. To further investigate the functional characterization of the *MRF4* gene, CRISPR/Cas9 gene editing was employed to produce MRF4 mutant progeny of Nile tilapia, allowing for the observation of expression levels of MRF genes and other growth-related genes in wild-type and gene-edited populations.

## 2. Materials and Methods

### 2.1. Experimental Fish and Husbandry

Adult Nile tilapia were collected from a commercial tilapia aquaculture farm (Docheon Fish Farm, Changnyeong-gun, Gyeongsangnam-do, Republic of Korea) and transported to the Laboratory of Molecular Physiology of the Department of Fisheries Science, Chonnam National University, Yeosu, Republic of Korea. The collected fish were acclimatized in rearing tanks for a week with continuous aeration. The water temperature of the rearing tanks were maintained at 26 °C. Fish were fed with commercial tilapia pellet feed. After acclimatization, Nile tilapia were used in different experiments.

### 2.2. Identifying Myogenic Regulatory Factor 4 (MRF4), Bioinformatic, and Expression Analysis

#### 2.2.1. Identification of Myogenic Regulatory Factor 4 (MRF4) Gene in Nile Tilapia

The Nile tilapia *myogenic regulatory factor 4* (*MRF4*) gene (NCBI accession No. NM_001282891) was identified in the NCBI genome assembly (O_niloticus_UMD_NMBU) of Nile tilapia using BLAST search.

#### 2.2.2. Tissue Collection for Gene Cloning

Ten Nile tilapia were anesthetized using tricaine methanesulfonate (MS-222) before tissue collection. Skeletal muscle tissues were then collected for cloning and sequencing of the *MRF4* gene. The collected tissues were immediately flash frozen in liquid nitrogen and stored at −80 °C.

#### 2.2.3. Total RNA Extraction and cDNA Synthesis

Total cellular RNA was extracted from the collected tissues using the ISOSPIN Cell and Tissue RNA kit (Nippon Gene, Tokyo, Japan). First-strand and 5′ and 3′ rapid amplification of cDNA ends (RACE) cDNAs were synthesized from total RNA using the Superscript III First-strand cDNA synthesis kit (Invitrogen, Waltham, MA, USA) and the SMARTer^®^ RACE 5′/3′ kit (Takara Bio Inc., Shiga, Japan), respectively.

#### 2.2.4. Cloning of Full-Length Nile Tilapia *MRF4* (*NT-MRF4*) cDNA

The full-length cDNA sequence of *MRF4* was cloned following the method described previously by [33]. Initially, a partial fragment of the *NT-MRF4* gene was cloned using a Phusion^®^ High-Fidelity DNA Polymerase kit (New England Biolabs Inc., Ipswich, MA, USA) by reverse transcription-polymerase chain reaction (RT-PCR). Primers were designed based on the predicted genomic sequence of Nile tilapia. Next, 5′-RACE and 3′-RACE primers were designed from this partial fragment. The 5′-RACE and 3′RACE fragments were amplified using the SMARTer^®^ RACE 5′/3′ kit. The RACE PCR products were ligated into the linearized pRACE vector and transformed into stellar competent cells (Takara Bio Inc., Shiga, Japan). Positive clones were selected and sequenced at Macrogen (Seoul, Republic of Korea). The 5′- and 3′-RACE fragments were combined, with the overlapping region of the partial fragment trimmed to obtain the full-length cDNA sequence of *NT-MRF4*. The primers used for *NT-MRF4* gene sequencing are listed in Table 1, and the PCR thermal cycling conditions are provided in Supplementary Table S1.

#### 2.2.5. Bioinformatic Analysis of the NT-MRF4

The nucleotide and protein sequences of the *NT-MRF4* gene were analyzed using various online bioinformatic tools and software. The source information for all tools and software used in this study is listed in Supplementary Table S2.

##### Analysis of General Sequence Features

The open reading frame (ORF) of *NT-MRF4* was identified with **ORFfinder**. The amino acid sequence of *NT-MRF4* was predicted using the **EMBOSS Transeq** online tool. **ProtParam** was employed to calculate the molecular weight and other physicochemical properties of the NT-MRF4 protein. Conserved domains and functional motifs were analyzed through the **NCBI Conserved Domain** database, Simple Modular Architecture Research Tool (**SMART**), and **Motif scan**. The nuclear localization signal was predicted using **NLStradamus** web server. Gene Ontology (GO) terms were predicted using the Contact-guided Iterative Threading ASSEmbly Refinement (**C-I-TASSER**) server. The genomic exon–intron structure of *NT-MRF4* was predicted using the SCIPIO web tool.

##### Multiple Sequence Alignment

Seven amino acid sequences of MRF4 from different chordate species were aligned using the online multiple sequence alignment program **ClustalO** and subsequently visualized and edited using **Jalview** software v. 2.11. The full-length amino acid sequences of MRF4 from 7 species were obtained from the NCBI database, and comprehensive sequence details are presented in Supplementary Table S3.

##### Phylogenetic Analysis

A phylogenetic tree of the MRF4 was constructed using protein sequences from 25 chordate species with **MEGA** v. 11.0.8 software. The protein sequences were aligned using the **ClustalO** aliment option. The evolutionary phylogenetic tree was constructed using the neighbor-joining algorithm with the bootstrap method and 1000 replicates. The full-length amino acid sequences of MRF4 were obtained for 25 species from the NCBI database, and comprehensive sequence details are presented in Supplementary Table S4.

##### Synteny Analysis

A synteny map of MRF4 was generated using a web-based synteny browser **Genomicus ver. 110.01**. Genes flanking next to *MRF4* were obtained for six chordate species viz. Nile tilapia (*Oreochomis niloticus*), zebrafish (*Danio rerio*), frog (*Xenopus tropicalis*), chicken (*Gallus gallus*), mouse (*Mus musculus*), and human (*Homo sapiens*) from Genomicus, as mentioned previously by [34].

##### Structural Model Prediction of NT-MRF4 Protein

The two-dimensional structure of NT-MRF4 was predicted using the **PDBsum** online tool. The three-dimensional (3D) protein structure of NT-MRF4 was generated using the online protein structure and functional prediction program **I-TASSER**. Afterward, the generated 3D model was refined using the **GalaxyRefine** web server. The refined 3D model was then validated using the **MolProbity** server. The predicted 3D structure was visualized and analyzed using **UCSF ChimeraX** v.1.3 software.

##### Prediction of Subcellular Localization

The subcellular localization of the NT-MRF4 protein was predicted using **ProtComp** 9.0 and **CELLO** v.2.5 online tools, while interactive protein features were visualized through **Predict protein** and **Protter** servers.

##### Protein–Protein Interaction Network Analysis

The protein–protein interaction network analysis of NT-MRF4 was performed using **STRING** database.

#### 2.2.6. Quantitative Real-Time PCR (qRT-PCR) Analysis of NT-MRF4 mRNA

##### Tissue Sample Collection, Total RNA Extraction, and cDNA Synthesis

Prior to tissue collection, the fish were anesthetized with MS-222, after which ten different tissue samples were collected: skeletal muscle (MUS), brain (BRN), gill (GIL), heart (HRT), stomach (STM), intestine (INT), liver (LIV), kidney (KID), spleen (SPL), and gonads (GND). A set of tissue samples were also collected during embryonic and larval development at the following stages: fertilized egg (FtE), 1 day-post-fertilization (1-F), 2-day-post-fertilization (2-F), 1-day-post-hatching (1-H), 10-day-post-hatching (10-H), 30-day-post-hatching (30-H), 60-day-post-hatching (60-H), and adult (ADT). A third set of tissue samples was collected from starvation experiments, where fish were starved for 14 days and then re-fed. Skeletal muscle samples were collected on the 1st day of the experiment as the control (CNT), then on the 7th day (7D-S) and 14th day (14D-S) of starvation, and finally from refed (ReF) fish after one day of re-feeding. All collected tissues were washed using 1× PBS, immediately flash-frozen in liquid nitrogen, and stored at −80 °C until total RNA extraction. Total cellular RNAs and cDNAs were extracted and synthesized, respectively, as described in Section 2.2.3.

##### qRT-PCR Analysis

To quantify the relative mRNA expression of *NT-MRF4* in different experimental tissues of Nile tilapia, qRT-PCR was performed using a 2× qPCRBIO SyGreen Mix Lo-Rox kit (PCR Biosystems Ltd., London, UK) in a LightCycler^®^ 96 System (Roche, Mannheim, Germany) as described previously by [35]. The qRT-PCR was conducted using 10 μL of reaction mixture containing 5 μL of SyGreen Mix, 0.5 μL of each gene-specific forward and reverse primer (Table 1), 1 μL of cDNA, and 3 μL of molecular grade ultrapure water. All reaction mixtures were prepared in quintuplicate, constituting biological replicates. The thermal cycling included pre-incubation at 95 °C for 3 min, followed by 40 cycles of 95 °C for 15 s, 60 °C for 20 s, and 72 °C for 15 s. Data analyses were performed using the LightCycler^®^ 96 System software. The relative gene expression levels were calculated using the 2^−ΔΔCT^ method, with Nile tilapia housekeeping gene *EF1a* (GenBank accession no. AB075952) as an internal reference. The expression level in the gonad was considered as the reference value (set to 1), to which all other data were normalized.

### 2.3. CRISPR/Cas9 Knockout of NT-MRF4 Gene in Nile Tilapia

#### 2.3.1. Design and Preparation of Single-Guide RNAs and Cas9 Protein

The genomic sequence of *NT-MRF4* was obtained from the NCBI database. The target sites were identified in exon 1 using the **CRISPRscan** online tool (https://www.crisprscan.org/; accessed on 26 May 2023). Two single-guide RNAs (sgRNA) were designed to target the *NT-MRF4* gene (Table 2). The sgRNAs were synthesized using the AccuTool^TM^ gRNA Design and Synthesis service of Bioneer (Daejeon, Republic of Korea).

#### 2.3.2. Artificial Fertilization and Preparation of One-Cell Embryos

During the spawning season, eggs and sperm were collected from mature Nile tilapia using the stripping method, as described previously by [19], and were temporarily kept in clean dishes. Artificial fertilization was performed by gently mixing the eggs and sperm with water in a Petri dish. After fertilization, one-cell embryos were collected for microinjection of the sgRNA and Cas9 complex.

#### 2.3.3. Preparation of sgRNA and Cas9 Complex and Microinjection

The sgRNA and Cas9 complex were prepared in a 300 μL tube on ice for microinjection, achieving a final concentration of 250 ng/μL for sgRNA and 500 ng/μL for Cas9 just before microinjection as described previously by [20]. A 1% (*v*/*v*) solution of phenol red (Sigma-Aldrich, St. Louis, Mo, USA) was prepared and added to the microinject mixture to monitor the volume and position of microinjection. The one-cell embryos were microinjected with the *NT-MRF4* sgRNA-Cas9 complex using a nanoliter injection device (Nanoliter 2020 Injector, WPI, Sarasota, FL, USA). A total of 500 embryos were microinjected. Untreated embryos were used as the control (WT).

#### 2.3.4. Mutation Analysis in Cas9/sgRNA Microinjected Offsprings

Microinjected embryos were maintained in incubators, and the survival rate was determined after hatching. Body weight (g) and total length (cm) of MRF4 gene-edited (GE) and WT population were measured fortnightly. Fin samples of GE and WT Nile tilapia were collected from 3-month-old offspring to extract genomic DNA. Genomic DNAs were extracted using the AccuPrep Genomic DNA Extraction kit (Bioneer, Daejeon, Republic of Korea) following the manufacturer’s protocol. A 402 bp region containing the sgRNA target site was amplified using gene-specific primers (Table 2) and then sequenced for mutation analysis. Mutation analyses were performed using the **ICE** analysis tools of the **Synthego** webserver.

#### 2.3.5. Downstream Gene Expression Analysis in *NT-MRF4* Gene-Edited Nile Tilapia

##### Tissue Sample Collection, Total RNA Extraction, and cDNA Synthesis

Skeletal muscle tissues were collected from WT and GE Nile tilapia. The samples were collected and processed as described in Section 2.3.1. Total cellular RNAs and cDNAs were extracted and synthesized, respectively, as described in Section 2.2.3.

##### qRT-PCR Analysis

To quantify relative mRNA expression of the MRF genes (*MRF4, MyoG, MRF5, MyoD*) and myocyte enhancer factor 2 (MEF2) genes (*MEF2a*, *MEF2b*, *MEF2c*, and *MEF2d*) in WT and GE Nile tilapia, qRT-PCR was performed as described in Section 2.2.6. The primers used for qRT-PCR analysis are presented in Table 3.

### 2.4. Statistical Analysis

The mRNA expression values in the experimental tissues were expressed as the mean ± standard error of the mean (SEM). Changes in relative mRNA expression levels among the different tissues were statistically analyzed either by one-way analysis of variance (ANOVA) followed by Tukey’s post hoc test or Student’s *t*-test, as appropriate, using GraphPad Prism 10.1.1 software. Statistical significance was considered at *p* < 0.05. All graphs were prepared using GraphPad Prism 10.1.1 software.

## 3. Results

**Figure 1:**
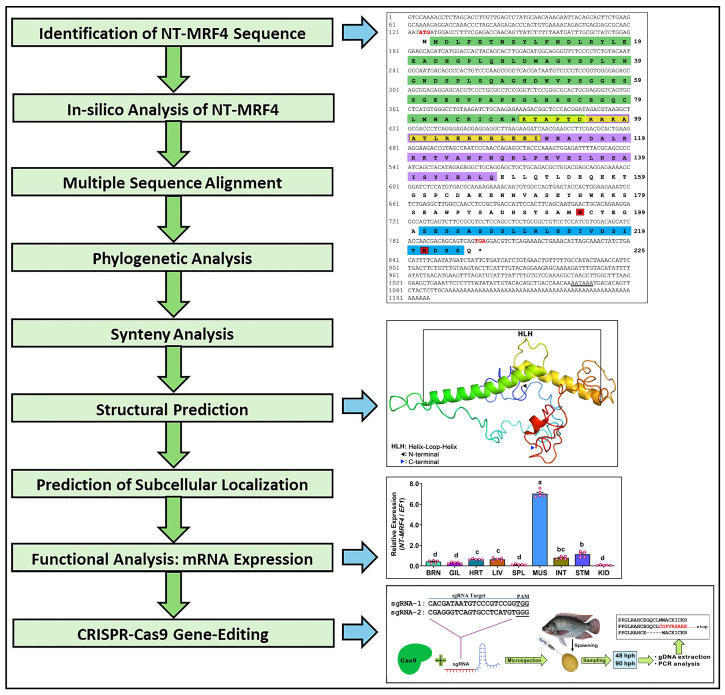
Complete work-flow of the study along with key results.

A comprehensive work-flow along with key results is presented in Figure 1.

### 3.1. General Features and Domains of NT-MRF4

**Figure 2:**
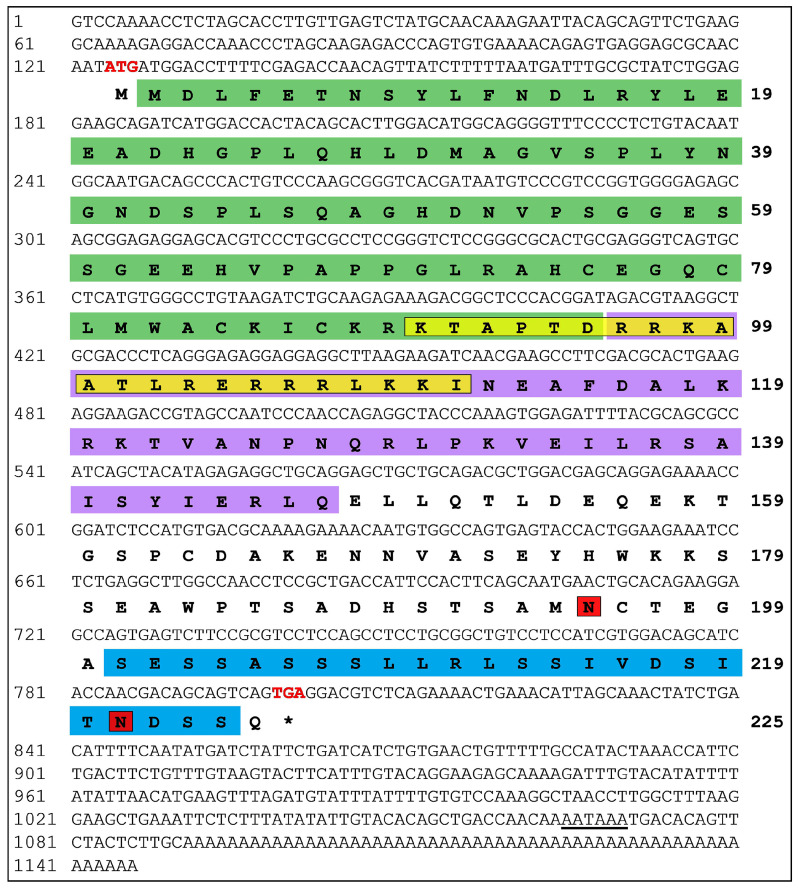
The full-length nucleotide and amino acid sequences of Nile tilapia MRF4 (NT-MRF4) gene (GenBank accession no. PQ497691). The numbers on the left and right represent the nucleotide and amino acid positions, respectively. The start codon (ATG) and stop codon (TGA; *) are shown in bold red letters. The putative polyadenylation signal (AATAA) is underlined. The myogenic basic domain is highlighted in green shading, and the helix-loop-helix (HLH) domain is shaded in violet. The overlapping nuclear localization signal is enclosed in a yellow box, while the serine-rich region is shaded in blue. The N-glycosylation sites are marked with red boxes.

A full-length nucleotide sequence of *MRF4* was cloned from Nile tilapia and designated as Nile tilapia *MRF4* (*NT-MRF4*). The *NT-MRF4* cDNA sequence (GenBank Accession No. PQ497691) was 1146 bp long and included a poly-A tail (Figure 2). The 5′ untranslated region (5′-UTR) was 123 bp and 3′-UTR was 345 bp. A putative polyadenylation signal (AATAAA) was located at 262 bp downstream of the stop codon. The *NT-MRF4* open reading frame (ORF) was 678 bp and encoded a putative protein of 225 amino acids (aa). The amino sequence of NT-MRF4 comprises a myogenic basic domain located at 2–95 aa, a helix-loop-helix (HLH) domain at 96–147 aa, and a serine-rich region at 201–224 aa. Additionally, the NT-MRF4 protein contains a nuclear localization signal (KTAPTDRRKAATLRERRRLKKI) located at 90–111 aa (Figure 2). The theoretical molecular weight (MW) and isoelectric point (pI) of NT-MRF4 were 24.91 kDa and 5.83, respectively. The total number of positively charged residues (Arg + Lys) and negatively charged residues (Asp + Glu) were found to be 32 and 27, respectively. The protein half-life computed was found to be 30 h in mammalian reticulocytes (in vitro). The grand average of hydropathicity (GRAVY) was—0.053. The negative value of GRAVY indicates that the protein contains a higher proportion of polar amino acids. The molecular formula of the protein was identified as C_1063_H_1697_0N_317_O_353_S_11_. Other additional physiochemical characteristics and detailed amino acid composition of NT-MRF4 protein are presented in Table 4. The genomic structure of *NT-MRF4* contains four exons and three introns spanning 3937 bp from start to stop codon (Supplementary Figure S1).

### 3.2. Multiple Sequence Alignment and Identity Index of NT-MRF4

**Figure 3:**
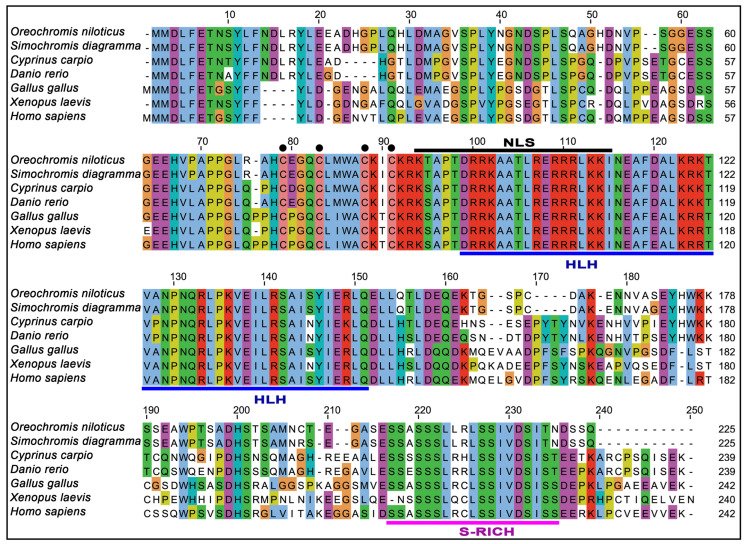
Multiple sequence alignment of MRF4 amino acid sequences of seven vertebrate species. The conserved HLH domain and S-rich region are marked with blue and pink underlines, respectively, while the nuclear localization signal (NLS) is marked with a black over line. Four conserved cysteine residues in the sequence alignment are indicated with black solid circles.

The multiple sequence alignment of NT-MRF4 was performed to identify conserved regions, reveal evolutionary relationships, and predict functional domains across species. The analysis revealed that the MRF4 protein in vertebrates contained a conserved helix-loop-helix (HLH) domain, a nuclear localization signal, and a conserved serine-rich region (Figure 3). The study also showed that four cysteine residues were conserved in the myogenic basic domain. A percent identity index of the NT-MRF4 protein with different fish species presented in Supplementary Table S5 demonstrated that NT-MRF4 shares over 95% identity with MRF4 proteins from Cichliformes fishes.

### 3.3. Phylogenetic Analysis

**Figure 4:**
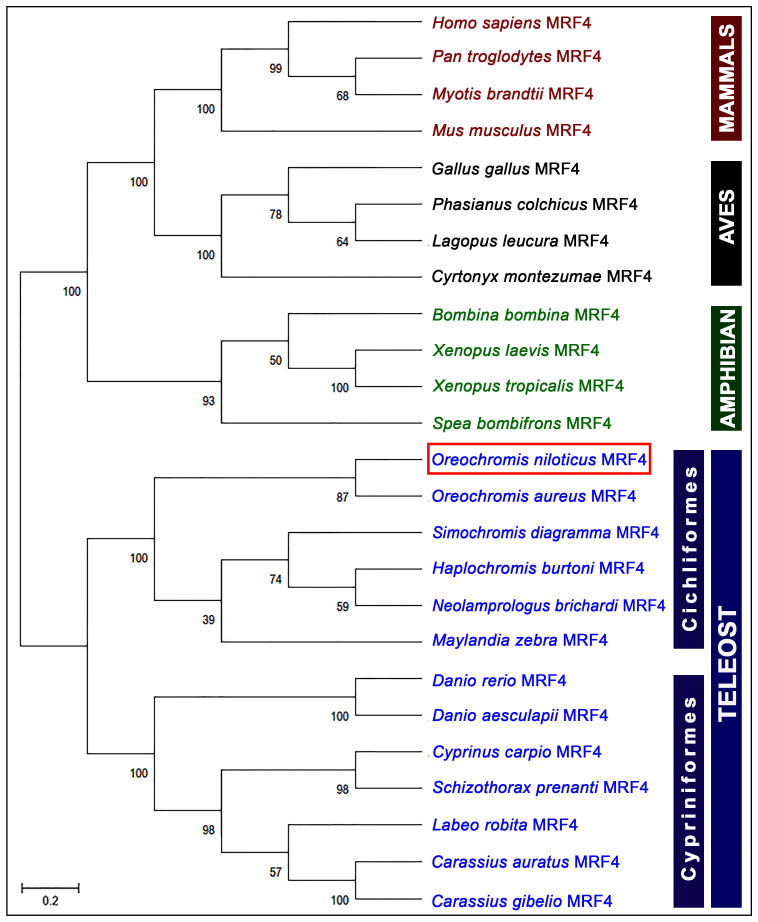
Phylogenetic tree of vertebrate MRF4 amino acid sequences. The analysis used the neighbor joining method with a bootstrap test of 1000 replicates. The numbers displayed at the nodes represent the bootstrap probability, with the scale bar corresponding to 0.2 units of the anticipated fraction of amino acid substitutions (where 1.0 units equals 100 PAMs).

An evolutionary phylogenetic tree of the MRF4 protein sequences was constructed to assess the potential evolutionary connections between NT-MRF4 and the MRF4 proteins of other vertebrate species. The unrooted phylogenetic tree displayed four major clades, mammalian, avian, amphibian, and teleost. Within the teleost clade, MRF4 further sub-clustered into Cichliformes and Cypriniformes orders. As expected, NT-MRF4 was positioned within the teleost MRF4 and sub-grouped with the Cichliformes species. Additionally, NT-MRF4 was most closely aligned with the MRF4 of *Oreochromis aureus*, the phylogenetically closest match (Figure 4).

### 3.4. Evolutionary Synteny Analysis of NT-MRF4

**Figure 5:**
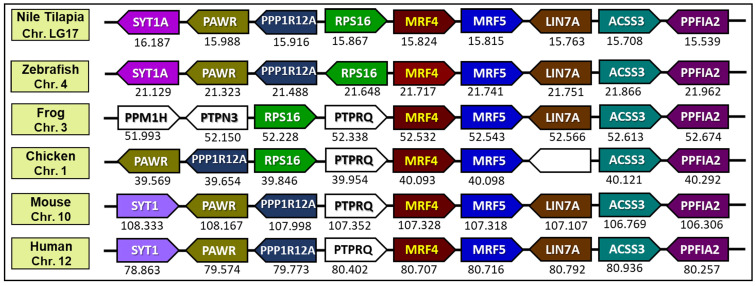
A synteny map compares orthologs of the MRF4 locus and the flanking genes in Nile tilapia and five other vertebrate species (zebrafish, frog, chicken, mouse, and human). The map was constructed using data obtained from Genomicus v. 110.01. The map displays genes as block arrows, orthologs of each gene in different species shown in the same column and color. The positions of gene (megabases, Mb) are presented below each block arrow based on the Ensembl database. Detailed chromosomal locations for these genes are provided in Supplementary Table S6.

After analyzing possible evolutionary connections of NT-MRF4 using phylogenetic analysis, a synteny analysis was performed to determine the origin and orthology relationship between NT-MRF4 with five other vertebrate species, including teleost (zebrafish), amphibian (frog), avian (chicken), rodent (mouse), and humans. The conserved synteny of *NT-MRF4* locus is located on Chr. LG17 in the genome of Nile tilapia (Figure 5), and indicates that this gene has a syntenic location. *NT-MRF4* is flanked by *MRF5* and *RPS16* on the right and left sides, respectively, which are also present in the teleost species. Following *MRF5*, *NT-MRF4* neighbors *LIN7A*, *ACSS3*, and *PPFIA2* on the right side. Meanwhile, *NT-MRF4* neighbors *RPS16*, *PPP1R12A*, *PAWR*, and *SYT1A* are on the left side. A similar gene neighboring pattern is observed in the teleost species, indicating that the *MRF4* is orthologous among teleost species. In other vertebrates, including humans, *MRF5*, *LIN7A ACSS3*, and *PPFIA2* are also found on the right side. Conversely, *PTPRQ* is present on the left side between *MRF4* and *RPS16* in frogs and chickens, while *PTPRQ* is located in the position of *RPS16* in mice and humans.

### 3.5. The Two- and Three-Dimensional Structure of NT-MRF4

**Figure 6:**
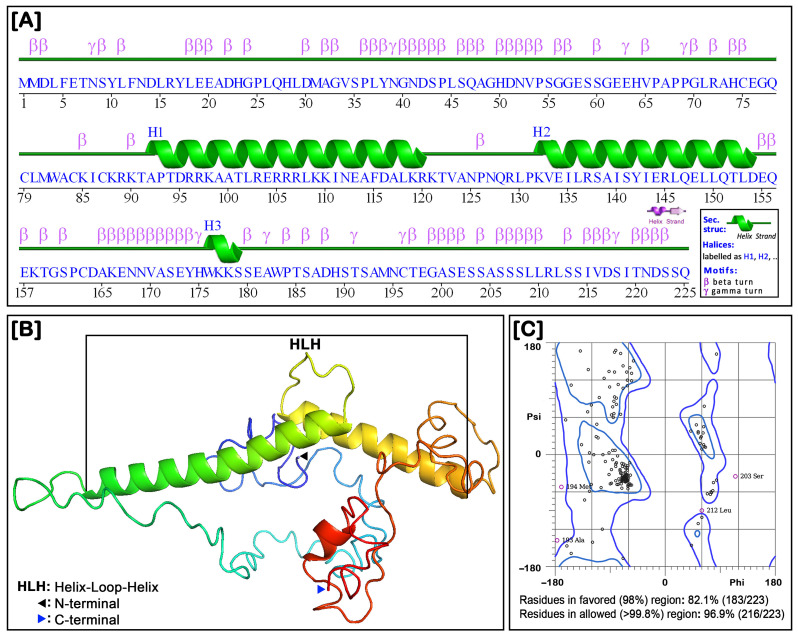
The two- and three-dimensional protein structures of NT-MRF4. (**A**) The two-dimensional structure showing the predicted strand-helix-coil arrangement, and the locations of β- and γ-turns in the NT-MRF4 sequence. (**B**) The three-dimensional structure of the NT-MRF4, with the HLH domain, N-terminal, and C-terminal indicated. (**C**) MolProbity Ramachandran plot validation analysis of the NT-MRF4 3D structure.

The structural model of NT-MRF was constructed to determine its structural conformation, which helps to understand its function, potential interaction, and role in the cellular process. The two-dimensional (2D) protein structure prediction revealed that the NT-MRF4 amino acid sequence contained three helices and four chains with 76 β-turns and 8 γ-turns in the chain region (Figure 6A). The three-dimensional (3D) structure of NT-MRF4 was characterized by a classical helix-loop-helix (HLH) configuration, consisting of three alpha helices interspersed by four loops (Figure 6B). Structure validation of the NT-MRF4 protein using the MolProbity server confirmed that 82.1% of the residues are in the favored region, and 96.9% are in the allowed region (Figure 6C).

### 3.6. Subcellular Localization of NT-MRF4

**Figure 7:**
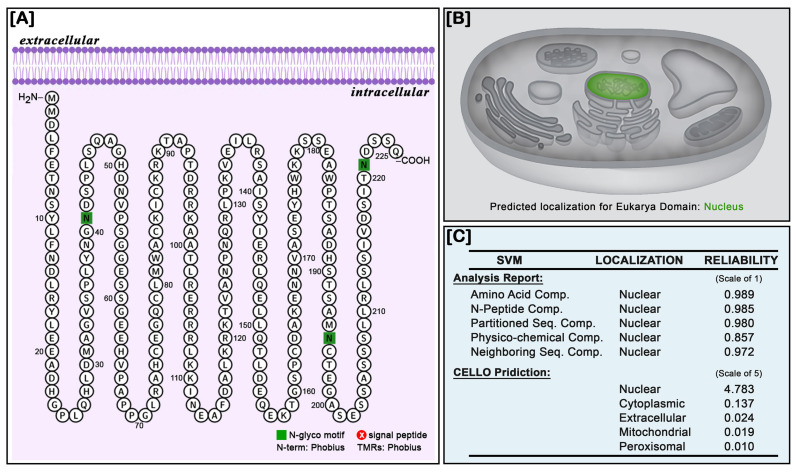
Subcellular localization of the NT-MRF4 protein. (**A**) The Protter server prediction of the NT-MRF4 protein indicates the location on the intracellular side of the cell membrane. (**B**) PredictProtein tool prediction identified NT-MRF4 as a nuclear-localized protein. (**C**) CELLO2 prediction algorithm defined NT-MRF4 as a nuclear localization protein.

The prediction of subcellular localization of NT-MRF4 was performed to identify its cellular location and infer its primary function. Both the Protter and PredictProtein servers predicted this protein to be localized in the nucleus (Figure 7A,B). The CELLO2 subcellular localization predictor, utilizing machine learning, also confirmed nuclear localization with a reliability score of 4.783 (Figure 7C).

### 3.7. Gene Ontology (GO) Analysis of NT-MRF4

The GO analysis was performed to predict the biological function, molecular role, and involvement in cellular processes of NT-MRF4. In the biological process (BP) category, NT-MRF4 was involved in “positive regulation of skeletal muscle fiber development” term (GO:0048743) with a C-score^GO^ of 0.50; “regulation of developmental process” term (GO:0050793) with a C-score^GO^ of 0.67 (Supplementary Figure S2). In the molecular function (MF) category, NT-MRF4 was associated with “transcription factor activity, RNA polymerase II distal enhancer sequence-specific binding” term (GO: 0003705) with a C-score^GO^ of 0.57 (Supplementary Figure S3A). Nonetheless, the cellular component (CC) category revealed its “nucleus” localization term (GO:0005634) with a C-score^GO^ of 0.89 (Supplementary Figure S3B).

### 3.8. Prediction of the Functional Protein–Protein Interaction Network of NT-MRF4

**Figure 8:**
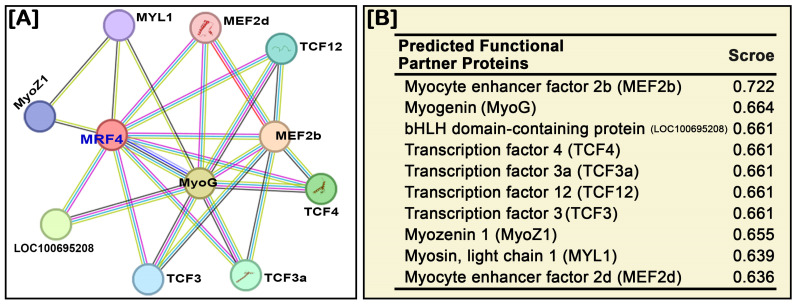
(**A**) The protein–protein interaction network of NT-MRF4 protein. Each node represents a protein, and each edge indicates interaction, either physical or functional. (**B**) Prediction score for functional partner proteins of NT-MRF4.

Protein–protein interaction network analysis was conducted using STRING ver.12.0 to identify and predict the potential functional protein interaction network of NT-MRF4 with other proteins. This analysis revealed a functional association of NT-MRF4 with ten proteins (Figure 8A). It was observed that MRF4 interacts with MyoG, myocyte enhancer factor two family proteins (MEF2b and MEF2d), and transcription factor (TCF) family proteins, which facilitate skeletal muscle development. The strongest interaction was predicted with MEF2b, with a score of 0.722, followed by MyoG, with a score of 0.664 (Figure 8B).

### 3.9. Relative mRNA Expression of NT-MRF4 in Different Experimental Tissues

**Figure 9:**
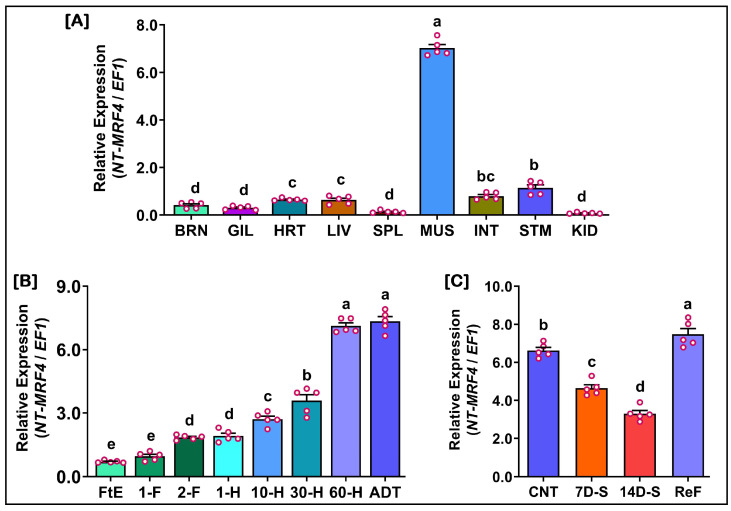
Relative mRNA expression levels of *NT-MRF4* in different experimental tissues of Nile tilapia. (**A**) Various tissues of Nile tilapia. BRN: brain, GIL: gill, HRT: heart, LIV: liver, SPL: spleen, MUS: muscle, INT: intestine, STM: stomach, KID: kidney. (**B**) Changes in *NT-MRF4* mRNA expressions during the development of Nile tilapia. FtE: fertilized egg, 1-F: 1-dpf, 2-F: 2-dpf, 1-H: 1-dph, 10-H: 10-dph, 30-H: 30-dph, 60-H: 60-dph, ADT: adult. (**C**) Changes in mRNA expressoins of *NT-MRF4* in muscle tissues from the starvation experiment. CNT: control, 7D-S: 7-day starvation, 14D-S: 14-day starvation, ReF: refed. In each bar graph, red circles represent individual data points, serving as biological replicates. Distinct letters above the bars indicate statistically significant differences (*p* < 0.05).

The relative mRNA expression of NT-MRF4 was analyzed in various tissues, at different ages of development and in the skeletal muscle tissues of starved Nile tilapia to investigate the potential role of the gene. The results are presented in Figure 9. The mRNA expression of NT-MRF4 in various tissues of Nile tilapia is presented in Figure 9A. Significantly higher mRNA expression was observed in muscle (MUS) tissue, while approximately seven-fold lower expression was observed in other tissues. Higher expression levels were observed among these tissues (other than MUS) in STM, followed by INT, HRT, and LIV. The MUS tissue was used in further mRNA expression experiments based on tissue-related expressions.

During the development of Nile tilapia, *NT-MRF4* mRNA expression levels significantly increased during the larval stages (dpf, days post-fertilization) compared to the embryonic stages (dph, days post-hatching). Expression levels continued to rise after hatching, reaching a peak at 60-dpf (Figure 9B). However, adult fish showed no significant changes in expression compared to those at 60-dpf.

In food-deprived (starved) Nile tilapia, *NT-MRF4* mRNA expression levels significantly decreased during the starvation period compared to the feeding phase (control and refeeding). Expression levels significantly increased during the re-feeding (ReF) phase compared to both the starvation period and the control (Figure 9C).

### 3.10. Generation of NT-MRF4 Mutant Progeny Using CRISPR/Cas9 and Mutation Analysis

**Figure 10:**
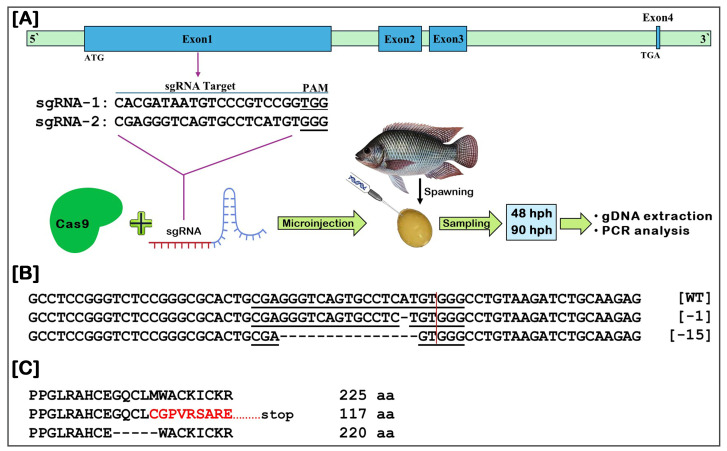
Generation and analysis of *NT-MRF4* disrupted Nile tilapia using CRISPR/Cas9 genome editing system. (**A**) Schematic diagram of CRISPR/Cas9 genome editing system followed in this experiment. (**B**) The mutations obtained in the *MRF4* gene-edited (GE) Nile tilapia. Deleted nucleotides in the MRF4 GE Nile tilapia are indicated with hyphens compared to wild-type (WT). (**C**) The predicted amino acid sequence of MRF4 in the GE and WT Nile tilapia. Deleted amino acids are indicated by hyphens, and altered amino acids are shown in red letters. The number on the right side represents the total number of amino acids in the coding region.

To disrupt *NT-MRF4*, two CRISPR/Cas9 target site sgRNAs (Table 2; Figure 10A) were designed within the coding region, located in exon 1. The Cas9 protein and sgRNA complex were then injected into one-cell embryos and reared until 90 dpf (Figure 10A). After hatching, no significant differences in aberration or mortality rates were observed between the control (WT) and the gene-edited (GE) microinjected groups (Supplementary Figure S4). Therefore, the dose of the sgRNA and Cas9 complex was considered suitable for the experiment.

Mutation analysis revealed that sgRNA1 did not cause any mutation, while sgRNA2 generated two types of deletions in the F1 generation: −1 bp and −15 bp (Figure 10B). The 15 bp deletion in the nucleotide sequence resulted in the loss of five amino acids. In contrast, the 1 bp deletion caused a frameshift mutation, leading to premature termination of transcription (Figure 10C). These results indicate that *NT-MRF4* sgRNA2 can effectively edit the *MRF4* gene in Nile tilapia, with mutation types and frequencies showing that most deletions caused frameshifts, leading to gene structure disruption. The sequencing chromatograms for these two individuals with the nucleotide deletions at the target site are presented in Supplementary Figure S5.

### 3.11. Change in mRNA Expressions of NT-MRF4 and Interacting Growth-Related Genes in WT and GE Nile Tilapia

**Figure 11:**
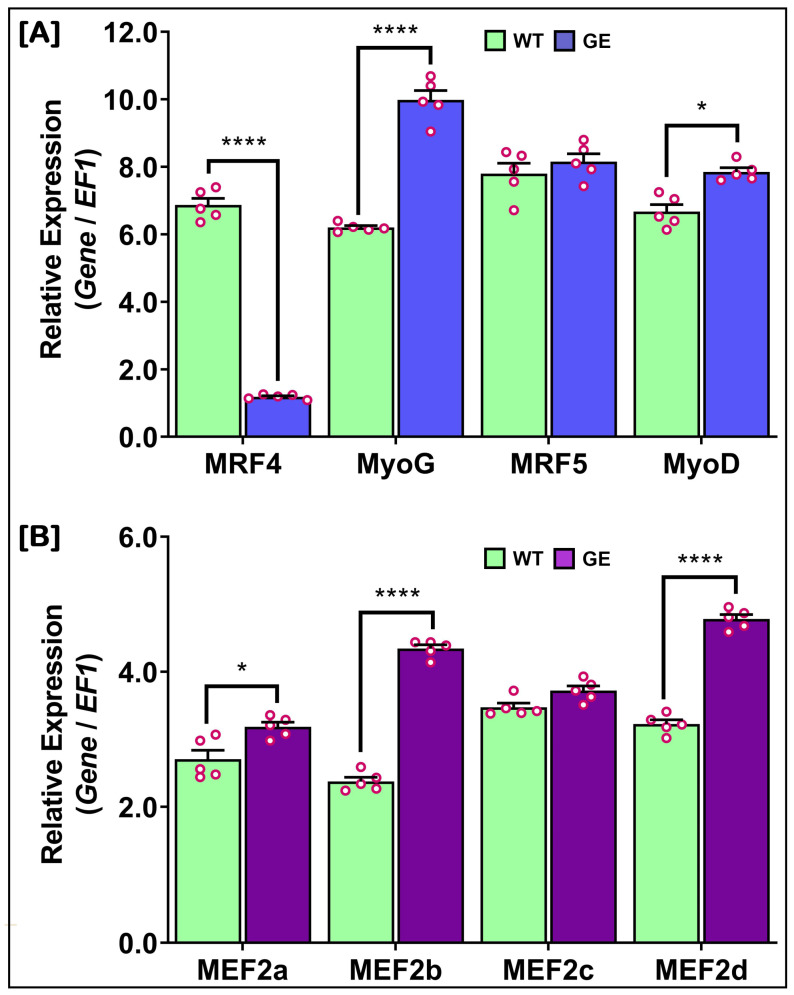
Changes in relative mRNA expression levels of *MRF* and *MEF2* genes in wild-type and *MRF4* gene-edited Nile tilapia. (**A**) Expressions of *MRF* genes. (**B**) Expressions of *MEF2* genes. In each bar graph, red circles represent individual data points, serving as biological replicates. Distinct asterisks above the bars indicate statistically significant differences (*p* > 0.05).

The relative mRNA expression analysis of *NT-MRF4* and interacting growth-related genes was performed in 90 dph fish to observe changes in the expression levels of *NT-MRF4* and interacting growth-related genes in WT and *NT-MRF4* GE Nile tilapia. The results are presented in Figure 11. The expression of *NT-MRF4* was significantly reduced in skeletal muscle tissue of GE Nile tilapia compared to WT. Interestingly, the expression of *MyoG* was significantly upregulated almost two fold in GE tilapia compared to WT (Figure 11A). Additionally, the expression of *MyoD* also significantly increased in GE Nile tilapia compared to WT. In contrast, *MRF5* expression showed no significant changes between GE and WT tilapia. The mRNA expression levels of *MEF2a*, *MEF2b*, and *MEF2d* were significantly upregulated in *MRF4* GE Nile tilapia compared to WT (Figure 11B). However, no significant differences were observed in the expression levels of *MEF2c* among WT and GE Nile tilapia.

## 4. Discussion

Myogenic regulatory factors (MRFs) play a pivotal role in the determination and differentiation of skeletal muscle, with MRF4 being the most highly expressed MRF in healthy mature muscles [11,16,36,37]. This is also significant for *MRF4*, as its expression undergoes marked upregulation during embryonic and larval development [17]. In the present study, a full-length sequence of *MRF4* was cloned from the muscle tissue of Nile tilapia, and functional characterization was performed using mRNA expression analysis and CRISPR/Cas9 gene editing.

As observed in other vertebrate species, the NT-MRF4 protein sequence contains a myogenic basic domain and conserved helix-loop-helix (HLH) domain [5,16]. This HLH domain is a characteristic feature of all MRF genes which is essential for DNA binding and transcription activation of muscle-related proteins [38,39]. Furthermore, NT-MRF4 contains a nuclear localization signal, which is a necessary feature for transcriptional factors. This observation is consistent with the predicted subcellular localization, which suggests the protein is localized in the nucleus. NT-MRF4 also contains a conserved C-terminal serine-rich region, a potential phosphorylation site that may play a role in regulatory activities [40]. The *NT-MRF4* sequence shows more than 95% sequence identity with Cichliformes fishes and more than 80% sequence identity with other fish species, indicating strong conservation in the length and constitution of the protein. Similarly, the pI of the deduced NT-MRF4 protein is acidic in nature as observed in other fish and higher vertebrates.

The multiple sequence alignment analysis of NT-MRF4 with selected vertebrate MRF4 revealed the presence of conserved domains (HLH, nuclear localization, and serine-rich) and residues across the amino acid sequences, which suggests evolutionary conservation of the HLH region. Phylogenetic analysis provided further insights into the evolutionary relationship of MRF4 proteins in vertebrates. Specifically, MRF4 was grouped into mammals, Aves, amphibians, and teleost clades. As expected, NT-MRF4 clustered within the teleost clade, subclustered with Cichliformes fish, and sub-grouped with *Oreochromis* species which share the closest phylogenetic relationship. Evolutionary synteny analysis also confirmed that Nile tilapia possess the *MRF4* gene, and *NT-MRF4* has a syntenic match with *MRF4* in other teleost species. Nile tilapia *MRF4* is flanked by genes encoding *MRF5* and *RPS16*, which are common genomic features in teleost *MRF4*. In contrast, in other vertebrates, such as mammals, Aves, and amphibians, *MRF4* is flanked by genes encoding *MRF5* and *PTPRQ*. This difference is likely to result from the 2R genome duplication event in vertebrates, where an ancestral *MRF* is duplicated in tandem to generate two vertebrate *MRF* genes, *MRF4*, and *MRF5*. A similar synteny map of *MRF* genes was also reported previously [12]. It has been reported that *MRF5* is the ancestral gene of the MRF family. *MRF4* likely emerged from *MRF5* via a gene duplication event at the same locus. Later, *MyoG* originated from *MRF4* via a further duplication event, although *MyoG* was subsequently positioned on a different chromosome. *MyoD* subsequently arose from *MRF5* through another gene duplication event on a third chromosome. Phylogenetic analysis concluded that these four *MRF* genes evolved from a single ancestral *MRF* gene due to gene duplication events and subsequent divergent mutations [41].

Regarding tissue-specific expression, *NT-MRF4* mRNA exhibited the highest expression levels in the muscle tissue. A similar expression pattern was also observed in several fish species, including zebrafish [14], yellowtail amberjack [15], common snowtrout [16], ya-fish [17], and golden mandarin fish (*Siniperca chuatsi*) [7]. The expression of *NT-MRF4* in adult muscle tissue suggests its involvement in the regulation of muscle. During embryonic and larval development, *NT-MRF4* expressions increase from 1-dpf to 60-dph, with the highest expression observed in the post-larval stages and adult fish muscles. These findings indicate that *NT-MRF4* is involved in adult muscle regulation and both embryonic and larval development. The elevated expression in the later stages of development suggests that *NT-MRF4* plays a significant role during this period when muscle fibers are more pronounced and in the maintenance of adult muscle. The expression of *MRF4* was also reported in the embryonic and larval development of various vertebrate species, including yellowtail amberjack [15], olive flounder (*Paralichthys olivaceus*) [42], Jinding ducks (*Anas platyrhynchos domestica*) [43], and African clawed frog (*Xenopus laevis*) [44]. In common snowtrout, *MRF4* expression was higher in adult fish muscle compared to younger fish [16], which aligns with the findings of the present study. This may be linked to a higher terminal differentiation rate in the myogenic process of muscle during the later stage of the life cycle [14].

In the context of starvation and re-feeding in Nile tilapia, the expression of *NT-MRF4* significantly decreased during the starvation period compared to the control group. The observed changes in mRNA expressions during the starvation phase may be attributed to the suppression of muscle proliferation and differentiation due to the absence of sufficient nutrients, which likely contributes to the reduction in *MRF4* expression levels. However, during the re-feeding phase, the expression increased significantly compared to the starvation period. This upregulation during re-feeding may be associated with the process of muscle regeneration and the realignment of muscle fibers, which are critical for recovery after a period of nutritional stress and starvation-induced degradation of myofilaments, as reported in common snowtrout [16], Dabry’s sturgeon (*Acipenser dabryanus*) [45], and African clawed frog [46]. The increase in *MRF4* expression during re-feeding supports the notion that MRFs play an essential role in muscle repair and regeneration following muscle atrophy induced by starvation [46]. Similarly to the findings in the present study, a sharp decrease in *MRF4* expression was observed in Dabry’s sturgeon during prolonged starvation [45]. This decrease may be due to the negative impact of reduced *MRF4* levels on muscle development, as it likely inhibits both the proliferation and differentiation of muscle cells.

MRF4-knockout mice exhibited no observable physical abnormalities at birth [18]. Furthermore, RNAi-mediated silencing of *MRF4* in mice led to an increase in myofiber size or myofiber hypertrophy [13]. Similarly, deletion of the *MRF4* gene in mice promoted a slight trend toward increased muscle fiber size [18]. Despite the reductions in *MRF4* mRNA or protein levels in these mutants, inactivation of the *MRF4* gene does not appear to cause defects in muscle development. In the present study, CRISPR/Cas9-mediated disruption of the *MRF4* gene was performed in Nile tilapia to produce MRF4 mutants. The designed sgRNA effectively targeted and disrupted the *NT-MRF4* gene, resulting in two forms of deletion. One deletion introduced a frame-shift mutation that generated a premature stop codon, causing early translation termination, and disrupting the molecular functions of the *NT-MRF4* gene. Meanwhile, gene editing of *NT-MRF4* led to a significant reduction in *NT-MRF4* mRNA levels in GE Nile tilapia compared to WT fish. Previous studies also reported that CRISPR/Cas9-mediated gene disruption lowers the mRNA levels of target genes in several fish species, including zebrafish [23] and mud loach [47].

The present study investigated the role of *MRF4* in regulating muscle-specific gene expression by examining the expression of *MRF* and *MEF2* genes in GE Nile tilapia. The relative expression levels of *MyoG* and *MyoD* were significantly increased in GE Nile tilapia compared to WT fish, while no significant changes were observed in *MRF5* expression. Previous studies have indicated that in *MRF4* knockout mice, *MRF4* function is likely compensated by other myogenic factor genes, such as *MyoG* and/or *MyoD*, allowing muscle development and maintenance to continue [18]. In the present study, among the *MEF2* genes, the expression of *MEF2a*, *MEF2b*, and *MEF2d* increased significantly in *MRF4* GE Nile tilapia, while no significant change was observed in *MEF2c* expression. These changes in expression help to continue the maintenance of muscle development in *MRF4* GE Nile tilapia. The protein–protein interaction network predictions for NT-MRF4 revealed a strong association with MEF2 (MEF2b and MEF2d) and MyoG proteins. Indeed, MEF2 proteins are known to regulate the function of myogenic bHLH genes, including MyoG and MRF4, and play a role in skeletal muscle development during embryogenesis and skeletal muscle maturation [48,49]. Additionally, it has been demonstrated that knockdown of *MRF4* increases the transcriptional activity of *MEF2*, while *MRF4* overexpression represses it. In contrast, *MyoG* overexpression has the opposite effect on *MEF2* genes [12,50]. Among the MRFs, *MyoG* and *MRF4* are essential myogenic factors involved in the later stages of myogenesis [51,52], and previous studies suggest that *MRF4* and *MyoG* can functionally substitute for one another during this process [41]. Therefore, the strong association between *MRF4* and *MyoG*, along with the higher expression of *MyoG* in GE fish observed in the present study, suggests that *MyoG* may compensate for the function of *NT-MRF4* to maintain and continue muscle development and regulation in Nile tilapia. Based on the findings, future research could focus on the compensatory role of *MyoG* in the absence of *NT-MRF4* and further explore the regulatory networks involving MEF2 family members in muscle development. Additionally, the use of CRISPR/Cas9 gene editing in non-model organisms like Nile tilapia could provide deeper insights into myogenic regulation.

## 5. Conclusions

*NT-MRF4*, a myogenic factor gene, was cloned from the muscle tissue of Nile tilapia, which plays an active role in muscle development and maintenance. *NT-MRF4* is exclusively expressed in the muscle tissue of adult Nile tilapia, as well as in embryonic and larval stages. Protein–protein interactions revealed a strong association of NT-MRF4 with MyoG and MEF2 proteins. CRISPR/Cas9 gene editing of *NT-MRF4* successfully generated two types of gene disruptions, resulting in a frame-shift mutation in the transcribed protein. Expression analysis of *MRF* and *MEF2* genes in GE Nile tilapia showed that *MyoG* compensates for the role of *MRF4* in gene-edited Nile tilapia, where *NT-MRF4* expression decreased drastically, and MyoG expression increased nearly double compared to WT fish. Additionally, the expression of *MEF2* genes, specifically *MEF2b*, *MEF2d*, and *MEF2a*, was significantly higher in GE Nile tilapia than in WT, which helps to continue muscle development in GE fish. Overall, these findings suggest that *NT-MRF4* regulates muscle development in Nile tilapia and that *MyoG* may compensate for *NT-MRF4* in GE Nile tilapia to maintain normal muscle development and growth.

## Figures and Tables

**Table 1:** Primers used for cDNA cloning and expression analysis of *NT-MRF4*.

Primer Name	Sequence (5′—3′)	Purpose
MRF4-Fw	GTACAATGGCAATGACAGCTC	RT-PCR
MRF4-Rv	CACTGGCTCCTTCTGTGCA	
NT-MRF4-5′RACE	GATTACGCCAAGCTTCTCTCCCCACCGGACGGGACATTATC	RACE PCR
NT-MRF4-3′RACE	GATTACGCCAAGCTTCAACCTCCGCTGACCATTCCACTTCAG	
NT-MRF4-Fw	TGGCAATGACAGCCCACTG	qRT-PCR
NT-MRF4-Rv	CTTACGTCTATCCGTGGGAG	
NT-EF1a-Fw	GGTGTGAAGCAGCTCATCG	
NT-EF1a-Rv	CACTGGTCTCCAGCATGTTG	

**Table 2:** sgRNA templates and mutation analysis of primers used in CRISPR/Cas9 experiment.

Primer Name	Sequence (5′—3′)	Accession No.	Purpose
sgRNA1	CACGATAATGTCCCGTCCGGTGG	NM_001282891	CRISPR/Cas9 target site
sgRNA2	CGAGGGTCAGTGCCTCATGTGGG		
MRF4-Mut-Fw	TTGCGCTATCTGGAGGAAGC		Mutation analysis
MRF4-Mut-Rv	CTCCTGCAGCCTCTCTATGT		

**Table 3:** qRT-PCR primers used for downstream gene expression analysis.

Primer Name	Sequence (5′—3′)	Accession No.	Length (bp)
MRF4-Fw	TGGCAATGACAGCCCACTG	PQ497691	178
MRF4-Rv	CTTACGTCTATCCGTGGGAG		
MyoG-Fw	TGTTGGAGTTGGAGTGACAG	GU246725	171
MyoG-Rv	CGTCTCTTCTCCCTCAGTGT		
MRF5-Fw	TCCAGTACATCGAGAGCCTG	XM_005456634	172
MRF5-Rv	CCGTTGCTGTAGTTTGCATTC		
MyoD-Fw	CAAGAGGAAGACGACCAACG	GU246715	170
MyoD-Rv	CGATGTAGCTGATGGCGTTG		
MEF2a-Fw	TCATGGACGAAAGGAACAGG	XM_025908678	170
MEF2a-Rv	CAGCAACACTTTGTCCATGTC		
MEF2b-Fw	GACCAGAGAAATAGACAGGTG	XM_005478988	160
MEF2b-Rv	GAACTTTGTCCATGTCTGTGC		
MEF2c-Fw	AGATCACGCGGATTATGGATG	XR_003213332	173
MEF2c-Rv	CTTGTCCATGTCTGTGCTGG		
MEF2d-Fw	CAGAGGATCACTGACGAACG	XM_025911272	171
MEF2d-Rv	GACCTTGTCCATGTCAGTGC		
EF1a-Fw	GGTGTGAAGCAGCTCATCG	AB075952	187
EF1a-Rv	CACTGGTCTCCAGCATGTTG		

**Table 4:** Physiological characteristics of the NT-MRF4 protein sequence.

Characteristics	Values	Amino Acid (aa) Composition			
		aa		No.	%
Number of amino acids	222	Alanine	(A)	20	8.9
Molecular weight (kDa)	24.91	Arginine	(R)	14	6.2
Theoretical isoelectric point (pI)	5.83	Asparagine	(N)	12	5.3
Total number of negatively charged residues (Asp + Glu)	32	Aspartic acid	(D)	13	5.8
		Cysteine	(C)	6	2.7
Total number of positively charged residues (Arg + Lys)	27	Glutamine	(Q)	8	3.6
		Glutamic acid	(E)	19	8.4
Atomic composition:		Glycine	(G)	11	4.9
Carbon (C)	1063	Histidine	(H)	7	3.1
Hydrogen (H)	1697	Isoleucine	(I)	7	3.1
Nitrogen (N)	317	Leucine	(L)	22	9.8
Oxygen (O)	353	Lysine	(K)	13	5.8
Sulfur (S)	11	Methionine	(M)	5	2.2
Formula	C_1063_H_1697_N_317_O_353_S_11_	Phenylalanine	(F)	3	1.3
Total number of atoms	3441	Proline	(P)	12	5.3
Estimated half-life (Mammalian reticulocytes, in vitro)	30 h	Serine	(S)	27	12.0
		Threonine	(T)	11	4.9
Instability index (II)	64.1	Tryptophan	(W)	3	1.3
Aliphatic index	68.18	Tyrosine	(Y)	5	2.2
Grand average of hydropathicity	−0.741	Valine	(V)	7	3.1

## Data Availability

The original contributions presented in this study are included in the article/Supplementary Materials. Few other data are not readily available because these data are part of ongoing research.

## Supplementary Materials

The following supporting information can be downloaded at: https://www.mdpi.com/article/10.3390/cimb46120820/s1, Table S1: PCR thermal cycling condition. (A) RT-PCR for partial sequencing and (B) RACE PCR; Table S2: The source information (web address) of all online bioinformatic analysis tools and software used in this research. Table S3: Sequence information of MRF4 genes used for multiple sequence alignment. Table S4: Sequence information of MRF4 proteins used for phylogenetic analysis. Table S5: Summary (CDS, % identity, MW, pI) of MRF4 genes in different fishes. Table S6: Chromosomal locations of gene orthologs in the synteny analysis of the MRF4 loci. All human gene names in the table are approved HGNC symbols. The genes in teleost, rodent, amphibian, and aves species have been given the name of their human orthologs and as mentioned in Genomicus. Chromosomal locations of genes are expressed in bp. Blanks represent genes that have not been found in the current genome databases. Figure S1: Genomic feature of *NT-MRF4* gene. (A) Genomic sequence of *NT-MRF4*. Blue-colored capital letters indicate exon sequences. (B) Exon-intron structure of *NT-MRF4*. Figure S2: Gene ontology (GO) of NT-MRF4. The figure illustrates the biological process (BP) components of GO. Figure S3: Gene Ontology (GO) of NT-MRF4. The figure illustrates the molecular function (A) and cellular component (B) components of GO. Figure S4: Aberration or mortality rates of Nile tilapia embryos after 1 dpf of Cas9 protein and sgRNA injection. Figure S5: The chromatograms represent the wild-type and mutant sequences, highlighting their respective nucleotide deletions (DNA with scissor in the sequence indicates cut site in nucleotides). The sequence above represents the sgRNA, with black-colored letters denoting the sgRNA and red-colored letters indicating the PAM sequence.
